# Global-key region collaborative noise-aware for electrocardiogram signal quality assessment

**DOI:** 10.1371/journal.pone.0355335

**Published:** 2026-08-07

**Authors:** Jijie Fan, Huihui Chang, Han Han, Jiawei Luo

**Affiliations:** 1 College of Land and Tourism, Luoyang Normal University, Luoyang, China; 2 School of Computer and Artificial Intelligence, Zhengzhou University, Zhengzhou, China; Mae Fah Luang University, THAILAND

## Abstract

Wearable electrocardiogram (ECG) devices enable long-term cardiac monitoring due to their convenience and comfort. However, various types of noise, such as baseline drift, power-line interference, and motion artifacts, can severely degrade signal quality and compromise the reliability of automated diagnosis. Consequently, ECG signal quality assessment (SQA) plays a crucial role in ensuring accurate downstream analysis. Existing supervised SQA methods rely heavily on large-scale annotated datasets, which are costly and time-consuming to obtain in real-world applications. Although unsupervised anomaly detection methods alleviate the dependence on labeled data, most existing approaches primarily focus on global signal characteristics and directly utilize reconstruction errors or feature-space distances for anomaly detection, while overlooking discriminative noise-related regions. As a result, their ability to distinguish noise contamination from physiological signal variations remains limited, leading to suboptimal generalization performance. To address this limitation, we propose a global-key region collaborative noise-aware ECG signal quality assessment method (NA-SQA) for unsupervised settings. NA-SQA integrates both global information and key noise-region features to enable more accurate quality evaluation. Specifically, we generate low-quality signals and corresponding quality labels by injecting various types of artificial noise into high-quality ECG signals. All signals are then passed through an autoencoder to reconstruct high-quality ECG signals. Subsequently, the original signals are used for global quality prediction, while the difference between the original and reconstructed signals is treated as the key noise region for key-region quality prediction. By jointly optimizing the global and key quality prediction tasks, the proposed model effectively integrates global information with more discriminative noise-region features, thereby enhancing its generalization capability to unseen low-quality ECG signals. Extensive experiments on four public ECG quality assessment datasets, namely BUTQDB, Icentia11K, EHOQA, and EAWQA, demonstrate the effectiveness of the proposed approach. NA-SQA achieves F1 scores of 91.42%, 79.62%, 81.59%, and 81.56% on the four datasets, respectively, consistently outperforming state-of-the-art unsupervised ECG quality assessment methods and demonstrating superior robustness and generalization capability for ECG signal quality assessment in real-world noisy environments.

## Introduction

In recent years, wearable electrocardiogram (ECG) devices have been widely used in dynamic cardiac monitoring due to their portability and wearing comfort, thereby promoting the development of long-term continuous ECG recording [1–3]. However, in real-world applications, complex and varying environments introduce various types of noise into ECG signals, such as baseline wander, power-line interference, and motion artifacts. These disturbances not only reduce signal usability but may also lead to misdiagnosis of arrhythmias, delayed treatment, or additional examinations, thereby increasing the medical burden and potentially compromising patient health [4–6]. Therefore, ECG signal quality assessment (SQA) plays a crucial role in ensuring signal reliability in wearable devices and remote monitoring. By leveraging automated techniques, ECG SQA can promptly identify and exclude low-quality signals, improve the accuracy of subsequent analysis, reduce clinical workload, optimize patient management, and enhance the efficiency of medical resource utilization.

With the rapid adoption of deep learning in medical signal processing, numerous deep learning-based ECG quality assessment methods have been proposed. For instance, Zhang et al. [7] integrated residual blocks with recurrent modules to extract features for three-class ECG quality classification; Huerta et al. [8] combined convolutional neural networks (CNNs) with oversampling-based data augmentation to enhance classification performance; Zhao et al. [9] proposed an improved frequency slice wavelet transform that converts ECG segments into two-dimensional time-frequency images, which are then classified by a CNN. Although these methods have achieved promising results, they are all supervised learning and therefore require a large amount of high-quality labeled data. However, the high cost of obtaining such labeled information hinders its large-scale application in practice.

In contrast, unsupervised anomaly detection usually does not require labeled anomaly samples, giving it a significant advantage and gradually becoming a new research hotspot [10]. Representative approaches include autoencoders [11], which learn to reconstruct input samples and identify anomalies based on reconstruction error; deep support vector data description (Deep-SVDD) [12], which constructs a minimal hypersphere in the feature space and detects anomalies according to the distance between features and the hypersphere center; the method proposed by Han et al. [13], which jointly models noise transformations and filtering transformations to detect anomalies from dual perspectives; and the approach proposed by Huang et al. [14], which incorporates pseudo-anomalous samples to assist DeepSVDD training and improve quality assessment performance. Although these methods have alleviated the dependence on labeled data to some extent, they usually focus on global features and lack in-depth exploration of key noise regions, thus limiting the improvement of generalization ability. To address this limitation, we further distinguish our method from conventional autoencoder-based residual anomaly detection. Unlike conventional autoencoder-based residual methods that directly use reconstruction error as an anomaly score, our method models the reconstruction residual as a key-region representation supervised by a unified quality-related label set. Under this supervision, the residual branch is guided not only by pseudo-noise labels but also by clean-signal labels, allowing the model to capture both prominent noise-induced fluctuations and subtle variations in high-quality signals. Furthermore, by jointly optimizing the key-region loss, the global prediction loss, and the consistency loss, the global branch provides overall quality context, the key-region branch focuses on local residual responses, and the consistency constraint enables their collaborative learning, thereby enhancing the model’s ability to distinguish noise-affected regions from subtle variations in high-quality signals.

To address the aforementioned issues, we propose a global-key region collaborative **N**oise-**A**ware method for ECG **S**ignal **Q**uality **A**ssessment (NA-SQA). Specifically, we first inject various artificial noises into high-quality electrophysiological signals to construct low-quality signals, while the noise type serves as an interference type label. All ECG signals are then passed through an autoencoder to reconstruct high-quality signals. The original signal is then fed into the prediction model to produce a global quality label. Meanwhile, the residual between the original and reconstructed signals is treated as the key noise region and passed through the prediction model to obtain a key quality label under the supervision of the global quality label. Jointly optimizing the prediction tasks for both global and key quality labels, enabling the model to focus on both global information and more discriminative key noise regions, thereby enhancing its generalization ability to unseen low-quality ECG signals.

The primary contributions of this paper are as follows:

- We propose a global-key region collaborative noise-aware ECG signal quality assessment method that simultaneously considers overall signal information and discriminative key noise regions, enabling more robust and generalizable quality evaluation.
- We propose a joint noise learning strategy that leverages global supervision to optimize the learning of critical noise regions, thereby fully capturing key robust features.
- Extensive experiments were conducted on several real-world ECG quality assessment datasets. The results demonstrate that the proposed method achieves state-of-the-art performance in quality evaluation, confirming the effectiveness of the model.

The remainder of this paper is organized as follows. The “Related Works” section reviews the existing literature relevant to ECG signal quality assessment and anomaly detection. The “Methods” section introduces the proposed NA-SQA framework in detail, including its overall architecture, key modules, and optimization objectives. The “Results” section presents extensive experiments on multiple datasets and provides comprehensive analyses of the obtained results. The “Limitations and Future Directions” section discusses the limitations of the current approach and outlines potential directions for future research. Finally, the “Conclusion” section summarizes the main findings of this work and highlights promising avenues for future investigation.

## Related Works

In reviewing existing ECG quality assessment studies, we found that categorizing prior work by noise type is neither practical nor representative of real-world conditions. Publicly available datasets rarely provide isolated single-noise scenarios, and real ECG recordings typically contain mixed and overlapping artifacts rather than clearly separable noise sources. As a result, noise-origin-based categorization does not meaningfully reflect how these methods are developed, evaluated, or applied in practice. Given these considerations, we organize the Related Work section according to methodological paradigms-traditional signal-processing approaches and deep-learning-based methods-rather than by specific noise categories. This structure more accurately captures the landscape of existing research and aligns with the broader objective of developing quality assessment models that generalize across diverse and unpredictable noise conditions.

### Traditional methods

Traditional methods for ECG quality evaluation mainly relied on statistical and morphological analyses. For example, Li et al. [15] introduced four quality indicators derived from power spectral analysis, QRS peak kurtosis, inter-detector agreement, and inter-lead consistency. Clifford et al. [16] extracted seven morphological and energy-based features and applied support vector machines for classification. In a more recent study, Kuetche F et al. [17] examined 39 signal quality metrics and identified 23 as the most robust for reliable ECG assessment. Andreotti et al. [18] used a Naive Bayes classifier with multiple SQIs to assess non-invasive fetal ECG quality. Rahman et al. [19] examined the robustness of various SQIs combined with classifiers such as MLP, Naive Bayes, LDA, and SVM. Liu et al. [20] developed an SVM-based model to distinguish acceptable from unacceptable ECG segments using typical SQIs. Nonetheless, these machine learning-based approaches generally show limited generalization and often perform poorly when confronted with complex cardiac rhythms such as atrial fibrillation, ventricular flutter, and ventricular tachycardia.

### Deep learning methods

Deep learning has shown a strong ability in extracting key features from ECG signals, enabling more reliable quality assessment. Current methods are mainly divided into supervised and unsupervised methods. Among supervised methods, some have focused on modeling temporal dependencies, such as Liu F et al. [21], who combined wavelet scattering transforms with LSTM networks. Menon K M et al. [22], who approximated QRS complexes using a Fourier series to measure correlations between ECG samples. Other approaches integrate multiple architectures to capture both key and global features. For example, Jin Y et al. [23] combined CNNs, bidirectional LSTM, and attention mechanisms, while Achinta M et al. [6] employed a lightweight 1D CNN based on Fourier amplitude spectra for efficient single- and multi-lead ECG quality assessment on resource-limited devices. Similarly, Liu Y et al. [24] segmented and compared QRS complexes to obtain robust feature representations.

Unsupervised anomaly detection [25] has attracted increasing interest because it does not rely on labeled abnormal data. Methods in this category include wavelet-based multi-feature reconstruction, as in Udit S et al. [26], and end-to-end convolutional autoencoders using reconstruction error and log-likelihood, as in Nick S et al. [27]. Domain-adaptive strategies, such as the lightweight CNN with MMD/CORAL used by Hui L et al. [28], have been proposed for interference detection. Xunhua H et al. [14] introduced pseudo-anomalyity augmentation combined with support vector data description (SVDD) to improve generalization. Reconstruction-based approaches are also widely employed, treating regions with high reconstruction error as anomalies [29–33]. Despite these advances, these methods only focus on global information and lack deep mining of key parts, so their generalization ability is still insufficient.

## Methods

**Fig 1:**
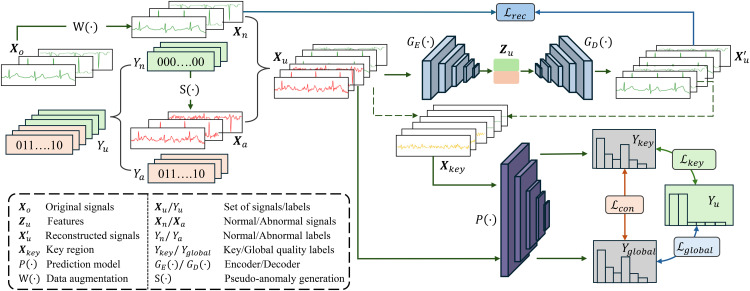
The proposed framework of NA-SQA.

In this section, we propose a global-key region collaborative noise-aware ECG signal quality assessment framework (NA-SQA). As illustrated in Fig 1, the overall framework primarily consists of data augmentation and pseudo-anomaly generation, a noise-aware reconstruction module, and a collaborative noise-aware learning module.

### Problem Statement

In this study, we formulate the ECG signal quality assessment task as an unsupervised anomaly detection problem, where high-quality ECG signals are defined as normal, and noise-contaminated low-quality signals are defined as abnormal. Our objective is to train solely on a set of high-quality ECG signals, denoted as $X_{o}=\{x_{o,i}{\}}_{i=1}^{N}$, where the *i*-th sample is represented as $x_{o,i}$. In this context, the original high-quality ECG signal set $X_{o}$ refers to ECG recordings with sufficient signal quality for diagnostic analysis, which may include both normal rhythm signals and signals with pathological rhythms (e.g., atrial fibrillation, premature ventricular contractions, and premature atrial contractions), provided their waveform quality remains acceptable for assessment. We aim to learn an encoder $G_{E}(\cdot )$, a decoder $G_{D}(\cdot )$, and a prediction model P(·) to enhance the model’s generalization capability to unseen low-quality ECG signals, thereby enabling more accurate ECG signal quality assessment.

### Data augmentation and pseudo-anomaly generation

Wearable devices have rapidly developed due to their convenience and comfort. However, in complex data collection environments, factors such as individual physiological differences, environmental changes, and improper user operation often cause the collected ECG signals to exhibit amplitude variations, signal inversion, and various types of noise. While amplitude changes and signal inversion do not compromise the diagnostic validity of the ECG, noise interference can significantly degrade its clinical diagnostic capability. To enhance the robustness and generalization of the model in real-world applications, we propose a data augmentation and pseudo-anomaly generation strategy for ECG signals, consisting of the following two components:

**Data Augmentation:** For a given set of normal signals $X_{o}=\{x_{o,i}{\}}_{i=1}^{N}$, we introduce two transformations. These transformations preserve the temporal structure and diagnostic information of the signals while enhancing the model’s robustness to amplitude and polarity variations. Amplitude Scaling: With a probability of $p_{a}$, the overall signal amplitude is randomly scaled within the range $\left[f_{min},f_{max}\right]$ to simulate amplitude variations caused by differences in electrode contact quality or individual physiological characteristics. Here, $f_{min}$ and $f_{max}$ denote the minimum and maximum amplitudes of the signal, respectively. Waveform Inversion: With a probability of $p_{w}$, the signal is inverted to achieve polarity reversal, simulating conditions such as reversed electrode placement.

We perform data augmentation on $X_{o}$ to obtain the normal signal set $X_{n}=\{x_{n,i}{\}}_{i=1}^{N}$, as formulated below:


$$\begin{array}{c} X_{n}=W(X_{o}) \end{array}$$ (1)

where W(·) represents data augmentation.

**Pseudo-anomaly generation:** For a normal signal set $X_{n}$, we randomly inject various types of noise: Gaussian, Rayleigh, gamma, exponential, Poisson, and uniform noise, as well as frequency modulation, amplitude modulation, and sinusoidal perturbations. This process simulates eight common real-world interference scenarios: power line interference, electrode contact artifacts, motion-induced noise, electromyographic interference, baseline drift, channel noise, combined noise sources, and device-related disturbances. The resulting noise-contaminated pseudo-anomaly signal set is denoted as $X_{a}=\{x_{a,i}{\}}_{i=1}^{N}$, and is defined as follows:


$$\begin{array}{c} X_{a}=S(X_{n}) \end{array}$$ (2)

where S(·) denotes the pseudo-anomaly generation.

During the pseudo-anomaly generation process, we define the ground-truth labels based on the presence or absence of injected noise. Specifically, the label set for normal signals is denoted as $Y_{n}=\{y_{n,i}{\}}_{i=1}^{N}$, while the label set for pseudo-anomaly signals is denoted as $Y_{a}=\{y_{a,i}{\}}_{i=1}^{N}$. Specifically, a multi-hot encoding scheme is adopted, where each dimension corresponds to a specific noise type. For a pseudo-anomaly signal $x_{a,i}$, the label $y_{a,i}=[y_{\left(a,i,1\right)},y_{\left(a,i,2\right)},...,y_{\left(a,i,M\right)}]$ is defined as


$$\begin{array}{c} y_{\left(a,i,j\right)}=\left\{ \begin{array}{cc} 1, & \text{if the }j\mathrm{-th noise type is injected into}\;x_{a,i}\\ 0, & \text{otherwise} \end{array}\right. \end{array}$$ (3)

where *M* is the total number of noise types. For a normal ECG signal $x_{n,i}$, the corresponding label vector is $y_{n,i}=[0,0,...,0]$, indicating the absence of any noise.

It is important to emphasize that the multi-hot labels are not manually annotated ECG quality labels. Instead, they are automatically derived from the pseudo-anomaly generation process based solely on the known types of injected synthetic noise. As such, no external supervision from the target ECG quality assessment task is introduced. These labels are only used as auxiliary self-supervised signals to facilitate noise-aware feature learning. Since the model is trained exclusively on high-quality ECG signals and does not require manually labeled low-quality samples, the proposed framework still follows the unsupervised anomaly detection paradigm.

Finally, we merge the normal and pseudo-anomaly signal sets along with their corresponding label sets, resulting in the signal set $X_{u}=X_{n}\cup X_{a}$ and the label set $Y_{u}=Y_{n}\cup Y_{a}$.

### Noise-aware reconstruction module

To extract interference signals from the original signals for constructing global-key region correlation learning, we propose a noise-aware reconstruction module, as shown in Fig??. During the model training phase, the input mixed samples $X_{u}$ are first mapped into a low-dimensional latent space by the encoder $G_{E}(\cdot )$, producing compact feature embeddings $Z_{u}=G_{E}(X_{u})$.

These embeddings are then passed through the decoder $G_{D}(\cdot )$ to reconstruct the time-domain signals, resulting in $X_{u}^{\prime }=G_{D}(Z_{u})$, where $X_{u}^{\prime }=X_{n}^{\prime }\cup X_{a}^{\prime }$ correspond to the reconstructed forms of the augmented signals and pseudo-anomalous signals, respectively.

Since our objective is not only to ensure accurate reconstruction of the augmented signals but also to guide the model to automatically suppress noise interference and focus on the underlying high-quality components during the reconstruction of pseudo-anomalous signals, we define the reconstruction loss as follows:


$$\begin{array}{c} {ℒ}_{rec}={\left\|X_{n}^{\prime }-X_{n}\right\|}_{2}^{2}+{\left\|X_{a}^{\prime }-X_{n}\right\|}_{2}^{2} \end{array}$$ (4)

where $X_{n}$ denotes the high-quality signal, which may include both clinically healthy ECG signals and ECG signals with pathological rhythms as long as their waveform quality is diagnostically acceptable. $X_{n}^{\prime }$ represents the reconstruction of $X_{n}$, and $X_{a}^{\prime }$ denotes the reconstruction of the pseudo-anomalous signal generated by our pseudo-anomaly generation strategy. Since each pseudo-anomalous sample $X_{a}$ is generated by injecting noise into its corresponding high-quality signal $X_{n}$, the two signals share the same underlying physiological morphology and rhythm structure. Therefore, constraining $X_{a}^{\prime }$ toward $X_{n}$ is intended to recover the original high-quality counterpart of the same ECG segment by suppressing the injected noise, rather than converting pathological ECG morphology into clinically healthy morphology.

Based on this design, this loss not only ensures that high-quality ECG signals can be accurately reconstructed but also encourages the reconstructed pseudo-anomalous signals to recover their corresponding high-quality counterparts. This allows the model to capture and suppress noise components while preserving the underlying physiological morphology of the input signal. Ultimately, this enhances the model’s discriminative and feature extraction capabilities in noisy environments, significantly improving the quality and robustness of ECG signal representation.

### Collaborative noise-aware learning module

To effectively establish collaborative perception from global information to key noise regions, we design a collaborative noise-aware module that leverages the ground truth labels, global prediction labels, and key noise prediction labels, thereby enhancing the model’s robustness and generalization capability. Specifically, based on the reconstruction method proposed in the previous section, we compute the difference between the reconstructed signal $X_{u}^{\prime }$ and the original signal $X_{u}$ to extract the residual representation relative to high-quality ECG signals.


$$\begin{array}{c} X_{\text{key}}=X_{u}-X_{u}^{\prime } \end{array}$$ (5)

this residual representation may arise from noise contamination, limitations of the reconstruction model, normal physiological variability, or morphological differences due to abnormal cardiac rhythms.

Rather than directly using reconstruction error to localize noise, our method employs a predictor to determine whether the residual representation is more likely associated with noise-induced fluctuations or normal physiological variations. The Prediction model P(·) extracts key features from $X_{\text{key}}$ and predicts the probabilities of each type of noise signal:


$$\begin{array}{c} Y_{\text{key}}=P(X_{\text{key}}) \end{array}$$ (6)

In general, noise contamination tends to produce larger and more irregular residual responses, whereas normal physiological variability or pathological rhythm-related morphology usually leads to relatively structured and stable residual changes.

At the same time, to capture noise information in low-quality ECG signals, we input the original signal $X_{u}$ into the same multi-head classifier P(·) to perform global noise type prediction:


$$\begin{array}{c} Y_{\text{global}}=P(X_{u}) \end{array}$$ (7)

Our optimization objective consists of three components based on the above prediction results. First, we minimize the error between the key noise prediction labels *Y*_key_ and the ground truth labels $Y_{u}$. With the supervision of the unified quality label set $Y_{u}$ and loss-function optimization, the model can learn to distinguish noise-related residuals from normal physiological or pathological morphology-related residuals, thereby reducing the risk of misclassifying clinically meaningful signal variations as noise.:


$$\begin{array}{c} {ℒ}_{\text{key}}=\|Y_{\text{key}}-Y_{u}{\|}_{2}^{2} \end{array}$$ (8)

Following, we directly perform global prediction on the augmented signals, where the predicted global noise labels *Y*_global_ are expected to be strongly correlated with the ground truth labels $Y_{u}$. We minimize the error between them to evaluate the overall impact of noise on the global signal characteristics. The loss function for this objective is defined as:


$$\begin{array}{c} {ℒ}_{\text{global}}=\|Y_{\text{global}}-Y_{u}{\|}_{2}^{2} \end{array}$$ (9)

Finally, since the key noise regions are extracted from the noisy signals, they are strongly correlated with the noise types. Therefore, we impose a consistency constraint between the key predictions and the global predictions. In particular, the key noise prediction labels *Y*_key_ and the global noise prediction labels *Y*_global_ should maintain semantic consistency. By minimizing the error between them, the model can more effectively integrate global information with discriminative key noise-region features, thereby improving its generalization capability for unseen low-quality ECG signals. The loss function is formulated as:


$$\begin{array}{c} {ℒ}_{\text{con}}=\|Y_{\text{global}}-Y_{\text{key}}{\|}_{2}^{2} \end{array}$$ (10)

Through the above collaborative modeling, our method can effectively achieve the complementarity and synergy between global and local information, demonstrating stronger robustness and generalization in more complex dynamic environments, thereby significantly enhancing the reliability of ECG signal quality assessment.

### Loss function and anomaly score

To enable the model to effectively integrate global information with discriminative noise-region features and enhance its generalization capability, we define the joint learning loss function as:


$$\begin{array}{c} {ℒ}_{\text{NASQA}}={ℒ}_{\text{key}}+{ℒ}_{\text{global}}+{ℒ}_{\text{con}}+{ℒ}_{rec} \end{array}$$ (11)

After training, NA-SQA can effectively capture both the critical ECG features and noise characteristics while fully leveraging the key and global information of the signal. The final anomaly score is computed as the mean of the predictions from the two modules, taking the maximum value across all noise types:


$$\begin{array}{c} S_{\text{core}}=\max(\frac{Y_{\text{global}}+Y_{\text{key}}}{2}) \end{array}$$ (12)

where a higher *S*_core_ value indicates a higher likelihood that the signal contains anomalous noise. The training algorithm of NA-SQA is provided in Algorithm 1.

**Algorithm 1 NA-SQA: Training Process**

**Input:** High-quality ECG signals ${𝐗}_{o}$, autoencoder $G(\cdot ,\theta )=\{G_{E},G_{D}\}$, multi-head classifier P(·), noise types $\{n_{j}{\}}_{j=1}^{m_{c}}$, augmentation probability *p*, amplitude scaling range $\left[f_{\min},f_{\max}\right]$

**Output:** Trained autoencoder *G* and classifier *P*, anomaly scores *S*_core_

1: **for** each epoch **do**

2:  ${𝐗}_{n},Y_{n}\leftarrow W({𝐗}_{o},p,f_{\min},f_{\max})$        ▷ Generate augmented high-quality ECG signals

3:  ${𝐗}_{a},Y_{a}\leftarrow S({𝐗}_{n},\{n_{j}{\}}_{j=1}^{m_{c}})$         ▷ Generate pseudo-anomalous signals

4:  ${𝐗}_{u}\leftarrow {𝐗}_{n}\cup {𝐗}_{a}$, $Y_{u}\leftarrow Y_{n}\cup Y_{a}$       ▷ Combine normal and pseudo-anomalous samples

5:  ${𝐙}_{u}\leftarrow G_{E}({𝐗}_{u})$             ▷ Encode input signals

6:  ${𝐗}_{u}^{\prime }\leftarrow G_{D}({𝐙}_{u})$             ▷ Reconstruct signals

7:  ${𝐗}_{\text{key}}\leftarrow {𝐗}_{u}-{𝐗}_{u}^{\prime }$             ▷ Extract key noise regions

8:  $Y_{\text{key}}\leftarrow P({𝐗}_{\text{key}})$             ▷ Predict key-region noise labels

9:  $Y_{\text{global}}\leftarrow P({𝐗}_{u})$             ▷ Predict global noise labels

10:  ${ℒ}_{\text{rec}}\leftarrow \|{𝐗}_{n}^{\prime }-{𝐗}_{n}{\|}_{2}^{2}+\|{𝐗}_{a}^{\prime }-{𝐗}_{n}{\|}_{2}^{2}$     ▷ Reconstruction loss

11:  ${ℒ}_{\text{key}}\leftarrow \|Y_{\text{key}}-Y_{u}{\|}_{2}^{2}$          ▷ Key-region prediction loss

12:  ${ℒ}_{\text{global}}\leftarrow \|Y_{\text{global}}-Y_{u}{\|}_{2}^{2}$        ▷ Global prediction loss

13:  ${ℒ}_{\text{con}}\leftarrow \|Y_{\text{global}}-Y_{\text{key}}{\|}_{2}^{2}$       ▷ Consistency loss

14:  ${ℒ}_{\text{NASQA}}\leftarrow {ℒ}_{\text{rec}}+{ℒ}_{\text{key}}+{ℒ}_{\text{global}}+{ℒ}_{\text{con}}$    ▷ Joint learning loss

15:  Update θ and parameters of *P* by minimizing ${ℒ}_{\text{NASQA}}$

16: **end for**

17: Compute anomaly score for each sample: $S_{\text{core}}\leftarrow \max\left(\frac{Y_{\text{global}}+Y_{\text{key}}}{2}\right)$

18: **return** Trained autoencoder *G* and classifier *P*, anomaly scores *S*_core_

## Results

### Experimental parameters

In this study, the proposed NA-SQA model was implemented in PyTorch and trained for up to 2000 epochs with a batch size of 128. An early stopping strategy was applied, terminating training if the F1 score did not improve for 50 consecutive epochs. The Adam optimizer with a fixed learning rate of 0.001 was used consistently across all datasets. To ensure a fair and rigorous comparison, we adopted the officially released implementations of all baseline methods. In addition to following the parameter configurations reported in their original papers, we further applied for each baseline model under our evaluation scenario. Grid search [34] is a widely used and effective strategy for parameter selection, covering commonly recommended ranges of key hyperparameters-including learning rate, latent dimensionality, regularization coefficients, and batch size-based on the guidelines provided in the original publications. For each method, the configuration yielding the best validation performance was selected for the final comparison. The detailed layer architecture of NA-SQA is summarized in Table 1.

**Table 1:** Implementation details of the proposed Unet_1D-based method. Conv1D represents the 1D convolutional layer, kernel/stride/padding are shown in parentheses, BN denotes Batch Normalization, and ReLU is the activation function.

Encoder $G_{E}(\cdot )$	
#1	Conv1D(inChannel, 6, k = 5, s = 1, p = 2)+BN + ReLU
#2	Conv1D(6, 6, k = 5, s = 1, p = 2)+BN + ReLU
#3	Conv1D(6, 6, k = 5, s = 1, p = 2)+BN + ReLU
#4	MaxPool1D(2) → Conv1D(6, 12, k = 5, s = 1, p = 2)+BN + ReLU
#5	Conv1D(12, 12, k = 5, s = 1, p = 2)+BN + ReLU
#6	MaxPool1D(2) → Conv1D(12, 24, k = 5, s = 1, p = 2)+BN + ReLU
#7	Conv1D(24, 24, k = 5, s = 1, p = 2)+BN + ReLU
#8	MaxPool1D(2) → Conv1D(24, 48, k = 5, s = 1, p = 2)+BN + ReLU
#9	Conv1D(48, 48, k = 5, s = 1, p = 2)+BN + ReLU
**Decoder $G_{D}(\cdot )$**	
#1	ConvTrans1D(48, 48, k = 8, s = 2, p = 3)
#2	Conv1D(24 + 48, 24, k = 5, s = 1, p = 2)+BN + ReLU
#3	Conv1D(24, 24, k = 5, s = 1, p = 2)+BN + ReLU
#4	ConvTrans1D(24, 24, k = 8, s = 2, p = 3)
#5	Conv1D(12 + 24, 12, k = 5, s = 1, p = 2)+BN + ReLU
#6	Conv1D(12, 12, k = 5, s = 1, p = 2)+BN + ReLU
#7	ConvTrans1D(12, 12, k = 8, s = 2, p = 3)
#8	Conv1D(6 + 12, 6, k = 5, s = 1, p = 2)+BN + ReLU
#9	Conv1D(6, 6, k = 5, s = 1, p = 2)+BN + ReLU
#10	LastLayer(6, inChannel)
**Prediction Model 𝒫(·)**	
#1	Conv1D(inChannel, 8, k = 4, s = 2, p = 1)+BN + ReLU + MaxPool1D(k = 3, s = 1, p = 1)
#2	Conv1D(8, 16, k = 4, s = 2, p = 1)+BN + ReLU + MaxPool1D(k = 3, s = 1, p = 1)
#3	Conv1D(16, 32, k = 4, s = 2, p = 1)+BN + ReLU
#4	Conv1D(32, 64, k = 4, s = 2, p = 1)+BN + ReLU+AdaptiveAvgPool1D(1)+Flatten
#5	Linear(64, Noisetype)+Sigmoid

### Metrics

All methods were thoroughly evaluated using three different random seeds, and the final results were reported as the average of these runs. Following common practice in previous studies, we adopted standard evaluation metrics, including **Precision**, **Recall**, and **F1** score, to assess the performance of quality evaluation. All experiments were performed on an Ubuntu 24.04 Server equipped with GeForce RTX 3090 Graphics Cards, an Intel(R) Xeon(R) CPU E5-2640 with a clock speed of 2.4 GHz, and a memory capacity of 256 GB.

### Dataset

To evaluate the performance of the proposed NA-SQA model, we conducted experiments on two well-known public datasets as well as two self-collected real-world 12-lead datasets. To ensure a fair comparison, we follow the commonly used data-splitting strategy in time-series anomaly detection and divide the ECG data into training, validation, and test sets with a ratio of 0.8:0.1:0.1 [35–37]. All compared methods were evaluated under exactly the same data partition, split ratios, evaluation metrics, and experimental settings, ensuring that relative performance comparisons among models are fair and not influenced by the data-splitting strategy. Detailed information on the dataset splits is provided in Table 2. Multi-lead ECG recordings were treated as single-lead data, because noise distribution across different leads is generally unstructured and not strongly correlated with spatial topology or specific lead positions; thus, splitting multi-lead recordings into single-lead signals does not significantly affect the effectiveness of noise detection. The goal of our model is to evaluate signal quality. That is, the high-quality samples that do not contain significant noise, which we define as normal samples, and the low-quality samples that contain significant noise, which we define as abnormal samples. It should be noted that the division of normal and abnormal samples is independent of the rhythm type of the sample itself, such as atrial fibrillation, ventricular tachycardia, or atrial/ventricular premature beats. For datasets with multiple quality labels, high-quality signals were regarded as normal samples, while all other labels were classified as abnormal samples.

- **BUTQDB** [38] is a database developed by the cardiology research team at the Department of Biomedical Engineering, Brno University of Technology, for assessing ECG signal quality. It contains 18 long-term single-lead ECG recordings along with corresponding three-axis accelerometer data from 15 subjects (9 female, 6 male), aged 21–83 years. The recordings were collected between August 2018 and October 2019 during routine daily activities (“free-living conditions”) using a mobile ECG and accelerometer system (Bittium Faros 180), with ECG signals sampled at 1,000 Hz and accelerometer signals at 100 Hz, and a minimum duration of 24 hours. Signal quality was annotated in three classes: class 1 indicates that all prominent waves (P, T, and QRS complexes) are clearly visible; class 2 indicates that some waves cannot be reliably detected, but QRS complexes remain detectable; and class 3 indicates that QRS complexes cannot be reliably detected, making the signal unsuitable for analysis. In this study, class 1 signals are defined as normal, classes 2 and 3 as abnormal, and all available data from this dataset were used.
- **Icentia11K** [39] contains continuous raw ECG signals from 11,000 patients, totaling approximately 2 billion annotated heartbeats. The signals were recorded using a fixed chest-mounted single-lead probe at 16-bit resolution and 250 Hz, with a maximum recording duration of two weeks. The average patient age was 62.2 ± 17.4 years. Each heartbeat type (normal, premature atrial contraction, premature ventricular contraction) and cardiac rhythm (normal sinus rhythm, atrial fibrillation, atrial flutter, noise, etc.) was annotated by 20 technicians, covering diverse beat and rhythm variations. In this study, the noise category is defined as abnormal, all other categories as normal, and all available data from this dataset were used.
- **EHOQA** contains 12-lead ECG recordings from 91 patients, collected using Holter monitors at 250 Hz and segmented into 10-second intervals. Each lead was assigned a quality grade from A to D based on waveform clarity and diagnostic usability, with A indicating high-quality signals suitable for diagnosis, and B-D representing medium to low quality. To ensure consistency, all experiments using EHOQA were conducted on single-lead data extracted from the 12-lead recordings. In this study, grade A signals are defined as normal, grades B-D as abnormal, and all available data from this dataset were used.
- **EAWQA** is a private, dynamic ECG quality assessment dataset collected using wearable ECG garments, including 12-lead recordings from 100 patients sampled at 250 Hz and segmented into 10-second intervals. Each lead was independently annotated with quality grades A-D, reflecting waveform visibility and diagnostic usability: Class A indicates all waveforms are clear; Class B includes 1–3 noisy beats with minimal impact on diagnosis; Class C has more than 50% recognizable waveforms, partially affecting diagnosis; and Class D consists of entirely unrecognizable waveforms, making the ECG signals unusable. To ensure consistency, all experiments using EAWQA were conducted on single-lead data extracted from the 12-lead recordings. In this study, grade A signals are defined as normal, grades B-D as abnormal, and all available data from this dataset were used.

**Table 2:** Statistics of the real-world datasets.

Dataset	Category	Train	Val.	Test	Total
BUTQDB [38]	Normal	14731	1841	1842	18414
	Abnormal	13983	1737	1737	17367
Icentia11K [39]	Normal	29474	3684	3685	36843
	Abnormal	6076	759	760	7597
EHOQA	Normal	37348	4597	4443	46388
	Abnormal	21260	2735	2889	26884
EAWQA	Normal	27629	3288	3504	34421
	Abnormal	14635	1992	1788	18415

### Baselines

To evaluate the robustness of the proposed anomaly detection method NA-SQA and the effectiveness of pseudo-anomalies, we designed three evaluation strategies: (1) an unsupervised approach that trains only on normal samples from the training set; (2) a self-supervised approach that treats genuine noise-free data as positive and uses the pseudo-anomalies generated in this study as negative (“P” label); and (3) a supervised approach that treats all genuine noise-free data as positive and all actual noisy data as negative (“R” label). Since most existing time series anomaly detection methods are supervised and differ in data partitioning, feature extraction, and experimental settings, they cannot be directly compared with NA-SQA. Therefore, we selected nine representative unsupervised anomaly detection methods for comparison to comprehensively assess the effectiveness of the proposed approach.

- **U-Net** [29]: A symmetric encoder-decoder CNN that captures context while enabling precise keyization, widely used for segmentation tasks.
- **AE-CNN** [30]: A convolutional autoencoder that detects anomalies based on reconstruction errors.
- **Dseep-SVDD** [12]: Learns a hypersphere around normal data, with anomalies measured by distance to the center.
- **Ganomaly** [32]: A GAN-based model with dual encoders enforcing feature and content consistency.
- **BeatGAN** [31]: GAN approach for heartbeat-level ECG anomaly detection using latent representations.
- **USAD** [33]: An adversarial autoencoder with one encoder and two decoders, providing stable training.
- **SLMR** [40]: Captures temporal dependencies across short and long sequences using masked representation learning.
- **MTAE** [13]: Combines noisy and filtered reconstructions, detecting anomalies via cumulative reconstruction error.
- **EQA-SVDD-P** [14]: An ECG quality assessment method that employs pseudo-anomaly generation and Support Vector Data Description (SVDD) to learn the boundary of normal ECG signals, enabling the discrimination between high- and low-quality ECG recordings.
- **EQA-SVDD-R** [14]: A variant of EQA-SVDD trained with real anomalous samples instead of pseudo anomalies, where SVDD is used to model ECG quality characteristics and distinguish high-quality ECG signals from low-quality ones.

### Comparative experiments

This section provides a comprehensive evaluation of the effectiveness of the proposed NA-SQA method for ECG quality assessment. In the experiments, we compared with EQA-SVDD, a self-supervised quality assessment method using pseudo-anomalies, and a supervised baseline trained on real noisy data. Table 3 presents the detailed experimental results of all methods across four datasets. The results indicate that NA-SQA outperforms all baseline methods on every dataset. Among the baselines, U-Net achieves relatively good performance, demonstrating the effectiveness of its signal reconstruction capability, but using only normal signals is insufficient for comprehensive ECG quality assessment. Furthermore, EQA-SVDD-P consistently outperforms EQA-SVDD-R on most datasets, validating the effectiveness of the generated pseudo-anomalies. NA-SQA learns key quality labels under the supervision of global quality labels. By jointly optimizing the prediction of global and key quality labels, the model not only captures overall signal information but also focuses on more discriminative noise regions, thereby more effectively detecting anomalous features. This mechanism enhances the model’s generalization ability to unseen anomalies and demonstrates superior discriminative performance in complex multi-lead dynamic environments.

**Table 3:** Anomaly detection performance of all methods. All values are percentages (%) and the best results are in bold.

Dataset	BUTQDB			Icentia11K			EHOQA			EAWQA		
	Pre	Rec	F1	Pre	Rec	F1	Pre	Rec	F1	Pre	Rec	F1
U-net [29]	85.69^†^	86.53^†^	86.11^†^	76.46	80.48	78.42	64.61^†^	65.59^†^	65.10^†^	62.05^†^	63.66^†^	62.84^†^
AE-CNN [30]	69.16^†^	69.94^†^	69.55^†^	39.04^†^	41.10^†^	40.04^†^	55.81^†^	56.66^†^	56.23^†^	41.08^†^	42.15^†^	41.61^†^
Deep-SVDD [12]	74.94^†^	75.71^†^	75.34^†^	50.83^†^	53.51^†^	52.14^†^	55.12^†^	55.96^†^	55.54^†^	45.55^†^	46.74^†^	46.14^†^
Ganomaly [32]	65.93^†^	66.57^†^	66.25^†^	35.95^†^	37.85^†^	36.88^†^	42.23^†^	42.88^†^	42.55^†^	27.52^†^	28.24^†^	27.87^†^
BeatGAN [31]	66.34^†^	66.99^†^	66.67^†^	44.71^†^	49.06^†^	45.85^†^	58.03^†^	59.03^†^	58.58^†^	44.71^†^	45.87^†^	45.28^†^
USAD [33]	55.25^†^	55.79^†^	55.51^†^	39.08^†^	41.14^†^	40.09^†^	55.69^†^	57.83^†^	57.38^†^	40.88^†^	41.95^†^	41.41^†^
SLMR [41]	77.08^†^	77.59^†^	77.33^†^	53.04^†^	54.69^†^	53.33^†^	56.94^†^	57.83^†^	57.38^†^	47.95^†^	49.30^†^	48.61^†^
MATE [13]	57.57^†^	57.76^†^	57.67^†^	36.68^†^	37.06^†^	36.87^†^	55.24^†^	56.37^†^	55.80^†^	34.78^†^	35.01^†^	34.89^†^
EQA-SVDD-P[14]	90.35	91.23	90.79	70.58^†^	74.30^†^	72.39^†^	73.26^†^	74.37^†^	73.81^†^	70.79^†^	72.63^†^	71.70^†^
EQA-SVDD-R[14]	89.97^†^	90.85^†^	90.40^†^	70.21^†^	73.90^†^	72.01^†^	76.79^†^	77.96^†^	77.37^†^	70.64^†^	72.48^†^	71.55^†^
**ours**	**90.97**	**91.86**	**91.42**	**77.62**	**81.71**	**79.62**	**80.98**	**82.21**	**81.59**	**80.53**	**82.62**	**81.56**

*Note:* † indicates a statistically significant difference between the baseline method and NA-SQA (p < 0.05).

To further verify whether the performance gains of NA-SQA are statistically meaningful, we conducted Welch’s t-tests based on the results of three independent runs. For each evaluation metric, NA-SQA was compared with the best-performing baseline on the corresponding dataset. Due to space limitations, the detailed p-values are not reported in the table. Instead, statistically significant differences (*p* < 0.05) are indicated by the symbol †. As shown in Table 3, most performance improvements achieved by NA-SQA are statistically significant. In particular, on the Icentia11K, EHOQA, and EAWQA datasets, NA-SQA significantly outperforms the strongest baseline across nearly all evaluation metrics. On BUTQDB, although the performance of EQA-SVDD-P is already highly competitive, NA-SQA still achieves the best overall results. These findings demonstrate that the observed improvements are not caused by random variations and confirm the effectiveness and reliability of the proposed method.

### Ablation studies

In this section, we conduct ablation studies on three datasets to evaluate the effectiveness of each component in the NA-SQA method. The specific settings of the ablation experiments are as follows:

- wo-*GR*: Compared with NA-SQA, this model removes noise awareness on the original signal and performs noise recognition using only the key noise regions.
- wo-*KR*: Compared with NA-SQA, this model removes key-region noise awareness from the reconstruction error and performs noise recognition only on the original signal.

To further assess the contribution of each component, we also conducted Welch’s t-tests based on the results of three independent runs. As shown in Table 4, removing either the global-region module or the key-region module leads to performance degradation. In particular, the performance drop caused by removing the key-region module is statistically significant across all datasets and evaluation metrics. This result highlights the importance of learning discriminative noise patterns from key regions. The removal of the global-region module also reduces performance, with significant differences observed on most datasets and metrics. These findings indicate that both modules contribute to the final performance. More importantly, the combination of global and key-region noise awareness provides complementary information, leading to more robust and effective ECG quality assessment.

**Table 4:** Anomaly detection performance of all methods. All values are percentages (%) and the best results are in bold.

Dataset	BUTQDB			Icentia11K			EHOQA			EAWQA		
	Pre	Rec	F1	Pre	Rec	F1	Pre	Rec	F1	Pre	Rec	F1
w/o-*GR*	90.69	91.58	91.13	75.25^†^	79.21^†^	77.18^†^	80.37	81.60	80.98	79.33^†^	81.40^†^	80.35^†^
w/o-*KR*	89.59^†^	90.46^†^	90.02^†^	73.92^†^	77.81^†^	75.81^†^	78.86^†^	80.06^†^	79.46^†^	75.11^†^	77.06^†^	76.08^†^
**NA-SQA**	**90.97**	**91.86**	**91.42**	**77.62**	**81.71**	**79.62**	**80.98**	**82.21**	**81.59**	**80.53**	**82.62**	**81.56**

*Note:* † indicates a statistically significant difference between the baseline method and NA-SQA (p < 0.05).

### Visualization experiments

**Fig 2:**
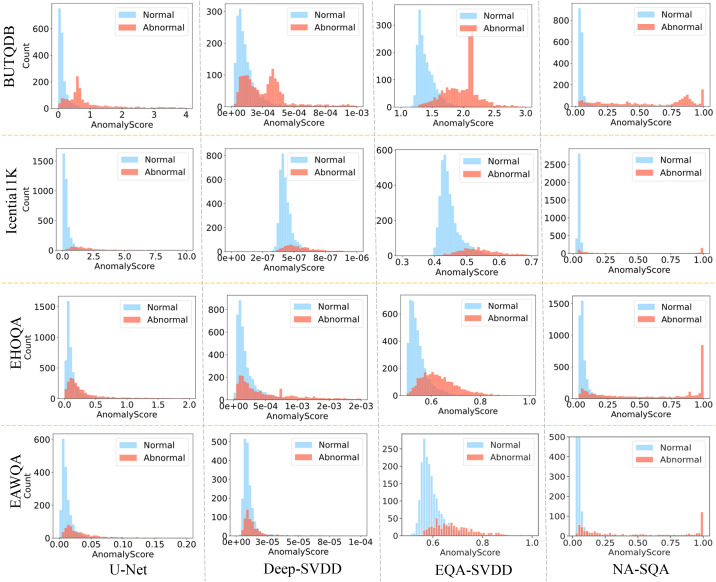
Visualization of noise score distributions of NA-SQA and compared methods on all datasets.

In this section, we visualize the distribution of anomaly scores for different methods across all datasets, as shown in Fig 2. The compared methods include strong baselines such as U-Net, Deep-SVDD, and EQA-SVDD.

The experimental results show that Deep-SVDD and EQA-SVDD can distinguish between normal and anomalous signals to some extent, demonstrating a certain capability for anomaly detection. However, their anomaly scores still lack clear decision boundaries, making it difficult to effectively identify hard-to-classify samples near the boundaries. In contrast, our proposed NA-SQA jointly learns from global and key noise regions and optimizes the feature extraction of key noise regions under the supervision of global quality labels, resulting in more distinct decision boundaries. This mechanism not only enhances the model’s ability to discriminate low-quality noisy signals but also improves the recognition of high-quality signals, thereby demonstrating stronger discrimination ability in multi-lead dynamic environments. As shown in the figure, anomaly scores of high-quality ECG signals are clearly clustered, further indicating that NA-SQA, while focusing on interference regions, also effectively captures the overall characteristics of the signals. These results fully validate the effectiveness of the proposed method in improving the generalization ability of quality assessment.

**Fig 3:**
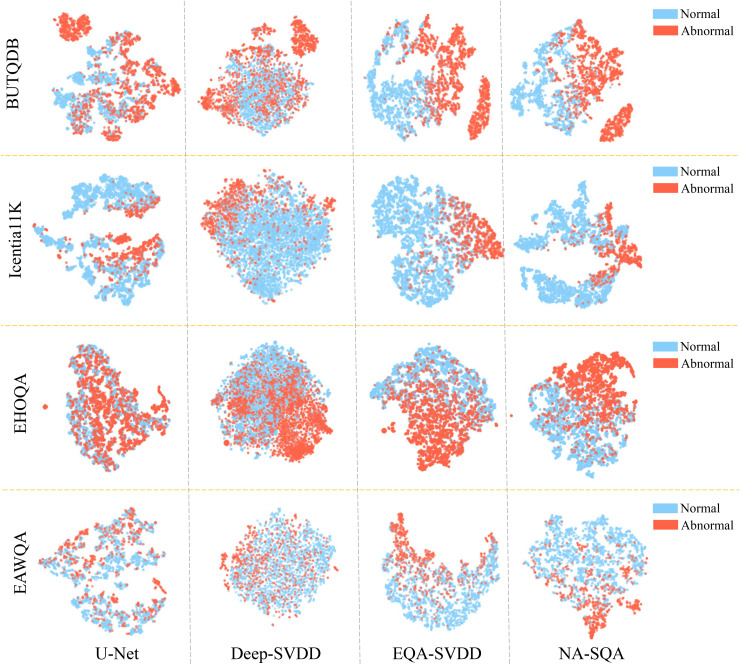
Visualization of feature distributions of NA-SQA and compared methods on all datasets.

In Fig 3, we present the feature embeddings obtained by different methods across all datasets. The learned feature embeddings are projected into a two-dimensional space using the T-SNE tool. In the figure, red and blue points correspond to noisy samples and high-quality samples, respectively. Our analysis shows that, across all datasets, NA-SQA outperforms other methods in distinguishing normal samples from anomalous ones. This is mainly attributed to NA-SQA’s joint learning between global and key noise regions, which enables better discrimination of the key features of noisy and high-quality signals, resulting in feature embeddings with clearer boundaries.

**Fig 4:**
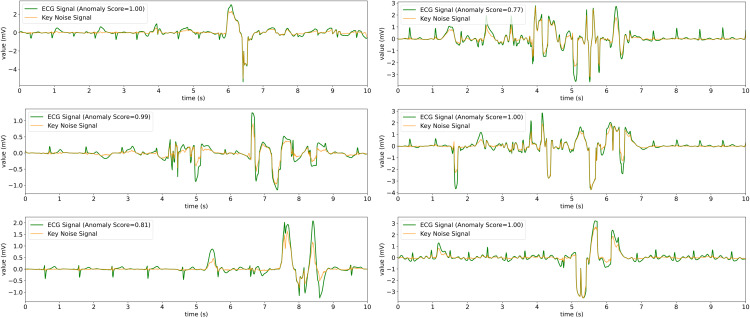
The visualization of key noise regions extracted by noise-aware reconstruction from real signals in a real low-quality dataset.

To verify the effectiveness of the proposed method under real interference conditions, we conducted a visualization experiment based on actual interference signals, as shown in Fig 4. Each subfigure simultaneously presents the original ECG signal (green) and the key noise signal (orange) extracted through reconstruction differences. The results show that the extracted key noise exhibits a high degree of consistency with the real interference signals in terms of waveform morphology, amplitude fluctuations, and temporal distribution, demonstrating significant positive correlation. The proposed noise-aware reconstruction module can effectively separate noise signals while complementing and collaborating with the original ECG signals, thereby enabling more robust ECG quality assessment.

### Pseudo-anomaly feature quality assessment

To quantitatively evaluate the quality of the generated pseudo-anomaly features, we employ the Kullback-Leibler (KL) divergence [42] to measure the discrepancy between different feature distributions. KL divergence is a widely used metric for comparing probability distributions, where a smaller value indicates greater similarity.

Specifically, latent features of real normal signals (E-N), real abnormal signals (E-A), and generated pseudo-anomaly signals (E-P) were extracted from the training set. The KL divergence between these distributions was then calculated, and the results are reported in Table 5. As shown, the KL divergence between E-A and E-P remains consistently low across all datasets. In contrast, the divergence between E-N and E-P is much larger, reaching 4.2511 on Icentia11K and 3.9644 on EAWQA. These results indicate that the generated pseudo-anomaly features are much closer to real abnormal samples than to normal samples in the feature space.

**Table 5:** Similarity measure results.

Dataset	BUTQDB [38]	Icentia11K [39]	EHOQA	EAWQA
**E-N ~ E-P**	1.2166	4.2511	1.6261	3.9644
**E-A ~ E-P**	0.3662	0.3914	0.4740	0.6439

**Fig 5:**
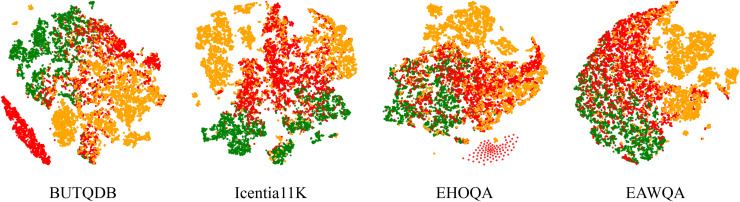
T-SNE scatter plot of real normal, real abnormal, and pseudo-anomalous feature distributions in the training set.

To further investigate the distribution characteristics of the generated features, we visualized different feature categories using t-SNE [43], as shown in Fig 5. Several observations can be made. First, the pseudo-anomaly features show substantial overlap with the real abnormal features, suggesting that the generated samples can effectively capture the characteristics of real anomalies. Second, the pseudo-anomaly features occupy a broader and more scattered region than the real abnormal features. This indicates that the generation process not only learns the underlying patterns of existing abnormal samples but also introduces additional variations. As a result, the coverage of the abnormal feature space is expanded. Such diversity is particularly beneficial for ECG signal quality assessment because low-quality ECG signals are often affected by various types of noise and exhibit highly heterogeneous characteristics.

### Computational analysis

To evaluate the deployment capability of the proposed method on wearable devices, we compare NA-SQA with several baseline methods in terms of model size, computational cost, and inference speed. All experiments are conducted under the same hardware and software environment. The input is a 10-s single-lead ECG signal sampled at 100 Hz. To simulate practical online inference, each ECG segment is processed individually. Model size is measured by the number of trainable parameters (Params). Computational cost is measured by floating-point operations (FLOPs). Inference time is obtained by averaging 1000 forward passes while excluding data loading overhead.

The results are presented in Table 6. Although NA-SQA requires slightly more computations than some baseline methods, the increase is limited. Meanwhile, NA-SQA still achieves fast inference. The average processing time for a single ECG segment is only 5.35 ms, which is much shorter than the data acquisition interval in wearable ECG monitoring systems. These results indicate that the proposed method can satisfy real-time processing requirements and has strong potential for deployment on resource-constrained wearable platforms.

**Table 6:** Computation analysis for NA-SQA with other methods.

Methods	Params/M	FLOPs/M	Testing time/ms
Unet [29]	0.06	17.65	4.32
Deep-SVDD [12]	0.02	4.39	2.25
EQA-SVDD [14]	0.04	12.93	1.49
NA-SQA	0.07	19.59	5.35

### Limitations and future directions

Despite the advantages demonstrated by NA-SQA, several limitations remain. First, NA-SQA relies on pseudo-anomaly generation to simulate low-quality ECG signals. Although these pseudo-anomalies improve training controllability and reduce dependence on manually annotated noisy data, the synthetic noise may not fully capture the complexity and diversity of real-world ECG artifacts. Second, the current framework primarily evaluates signal quality at the single-lead level, and its scalability to multi-lead spatial modeling remains to be investigated. Third, although NA-SQA shows promising performance in offline experiments, deployment in real-world wearable scenarios is constrained by device heterogeneity, computational resources, power consumption, and streaming inference requirements. Finally, clinically meaningful ECG morphology and nonlinear spectral characteristics are essential for downstream diagnosis [44,45], but the current model may not explicitly preserve these features during reconstruction and quality assessment. Future work will focus on improving the realism of pseudo-anomaly generation to better cover diverse noise types in real-world ECG recordings. We also plan to extend the framework to multi-lead spatial modeling to capture inter-lead correlations and improve assessment accuracy. Additionally, morphology-preserving constraints and diagnostic consistency evaluation will be incorporated to ensure clinically relevant waveform features are maintained [43,46]. Finally, strategies for real-time deployment on wearable devices with limited resources will be explored, aiming to enhance model robustness and practical applicability.

## Conclusion

This study proposes a global-key region collaborative noise-aware ECG quality assessment method (NA-SQA), designed to address the limitations of traditional approaches that cannot simultaneously capture global information and discriminative key noise regions, resulting in restricted generalization. NA-SQA employs data augmentation and pseudo-anomaly generation strategies to construct normal and pseudo-anomaly signals along with their corresponding quality labels. It uses global information for overall quality prediction, while capturing key noise-related residual representations based on the difference between the original and reconstructed signals, and performing key-region quality prediction under the guidance of global information. Rather than treating residuals as direct noise localization results, NA-SQA learns residual representations under the supervision of the unified quality label set $Y_{u}$. Since residuals may arise from noise contamination, reconstruction limitations, normal physiological variability, or abnormal rhythm-related morphology, this supervision helps the model distinguish irregular noise-induced fluctuations from relatively structured physiological or pathological variations, thereby reducing the risk of misclassifying clinically meaningful morphology as noise. This enables the model to focus on both global signal quality context and local degradation-sensitive responses, enhancing its generalization to unseen low-quality ECG signals. Extensive experiments validate the effectiveness of NA-SQA. This work can improve the accuracy of ECG quality assessment and provide support for the screening and analysis of wearable dynamic ECG data. Future work will focus on enhancing the fidelity of pseudo-anomaly generation and multi-lead spatial modeling, while incorporating morphological constraints to ensure clinical consistency, thereby bolstering the robustness and practical utility of the model.
